# Host and Pathogen Genetic Determinants of Brucellosis in Veterinary–One Health Interface: A Review

**DOI:** 10.3390/vetsci13080751

**Published:** 2026-07-29

**Authors:** Abdul Qadeer, Mohamed Tharwat, Ibrahim F. Halawani, Fuad M. Alzahrani, Khalid J. Alzahrani, Fahad A. Alshanbari, Muhammad Zahoor Khan

**Affiliations:** 1 Department of Medical Sciences, Shandong Xiehe University, Jinan 250109, China; qadeerabdul@sdxiehe.edu.cn; 2 Department of Clinical Sciences, College of Veterinary Medicine, Qassim University, P.O. Box 6622, Buraidah 51452, Saudi Arabia; atieh@qu.edu.sa; 3 Department of Clinical Laboratory Sciences, College of Applied Medical Sciences, Taif University, Taif 21974, Saudi Arabia; 4 Department of Medical Biosciences, College of Veterinary Medicine, Qassim University, P.O. Box 6622, Buraidah 51452, Saudi Arabia; 5 College of Agriculture and Biology, Liaocheng University, Liaocheng 252000, China

**Keywords:** brucellosis, genomics, transcriptomics, *Brucella* virulence genes, zoonosis, antimicrobial resistance, One Health

## Abstract

Brucellosis remains among the most widespread global zoonoses, yet its genetic architecture—spanning host susceptibility and pathogen virulence—is increasingly being investigated. Advances in whole-genome sequencing, transposon mutagenesis, CRISPR screening, and machine learning-driven transcriptomics have accelerated marker discovery on both sides of the host–pathogen interface, although most signatures remain at discovery or early-validation stages. In hosts, polymorphisms in innate immune receptors (*TLR2*, *TLR5*, *VDR*), cytokine regulators (*TNF*, *CTLA4*), and novel candidates such as *ASAP1* are linked to differential susceptibility in humans and livestock. In *Brucella*, functional genomics shows that virulence relies on interconnected regulatory circuits that control the type IV secretion system, lipopolysaccharide biosynthesis, and stress responses, with gene essentiality shifting according to the host immune context. Antimicrobial resistance may involve unconventional mechanisms, including metabolic and regulatory adaptation, that are not fully explained by classical resistance-gene paradigms and may implicate metabolic reprogramming and ribosomal modulation. This review synthesizes these converging lines of evidence, highlights key gaps—chiefly the scarcity of functional validation for host associations and limited multi-omics integration—and proposes a roadmap for translating genetic markers toward future diagnostic refinement and therapeutic target discovery, urging a shift from descriptive cataloging toward mechanistic and clinical validation.

## Simple Summary

Brucellosis imposes heavy economic burdens on livestock and poses a persistent zoonotic threat. Although infection rarely causes death, it commonly triggers abortion and stillbirth; however, the genomic and immunological drivers of this variable clinical outcome are largely unknown. This review brings together scattered findings from genetics, genomics, and transcriptomics to explain how both the animal host and the bacterium shape disease outcome. We highlight practical advances most relevant to veterinary practice: genetic markers that may contribute to breeding programs after independent validation to produce naturally resistant cattle, goats, sheep, and buffalo, and gene-expression signatures that may sharpen diagnosis and disease staging. We also clarify why antimicrobial resistance in *Brucella* may involve regulatory, metabolic, and chromosomal mechanisms that are not fully captured by classical acquired resistance-gene databases. By identifying where current evidence is strong, weak, or missing, this work offers researchers and practitioners key research priorities for advancing genetics-informed disease control, improving animal welfare, and reducing zoonotic transmission.

## 1. Introduction

Brucellosis is a globally significant zoonosis caused by facultative intracellular bacteria of the genus *Brucella*, widely distributed across Central Asia, the Middle East, the Mediterranean, Africa, South America, and parts of Eastern Europe, with Kazakhstan a significant hotspot [1,2]. Reported figures of roughly 500,000 notified human cases per year almost certainly understate the true burden due to widespread under-ascertainment and misdiagnosis; a recent modeling study that accounted for under-reporting estimated the actual global incidence at approximately 1.6–2.1 million new human cases each year [3]. The disease also causes devastating reproductive losses in livestock worldwide [4]. In humans, the infection typically presents after an incubation period of 2–4 weeks (ranging from several days to several months) with undulant fever, night sweats, fatigue, malaise, weight loss, headache, arthralgia, and myalgia [4]. Because these features are non-specific and mimic many febrile illnesses, the disease is frequently misdiagnosed; untreated infection may progress to a chronic, relapsing course with focal complications—most commonly osteoarticular disease (sacroiliitis, spondylitis, arthritis), but also neurobrucellosis, hepatosplenic involvement, and endocarditis, the leading cause of brucellosis-related death [4].

The genus *Brucella* comprises small, Gram-negative, non-motile coccobacilli that survive and replicate within host phagocytes [5,6]. *Brucella* species are classically differentiated by their preferred animal reservoirs: *B. melitensis* (sheep and goats), *B. abortus* (cattle), *B. suis* (swine), and *B. canis* (dogs) account for most human brucellosis cases, with *B. melitensis* being the most virulent in humans. Additional species, including *B. ovis*, *B. neotomae*, and the marine-associated *B. ceti* and *B. pinnipedialis*, expand the genus, while their highly conserved genomes complicate species-level discrimination and underscore the value of molecular characterization [7,8]. Among these, *B. melitensis* and *B. abortus* merit particular emphasis, as they account for the majority of livestock and human brucellosis and impose the heaviest economic burden. They likewise remain the most comprehensively characterized members of the genus at the genetic, genomic, and transcriptomic levels, whereas comparable evidence for the remaining species is sparse.

**Figure 1:**
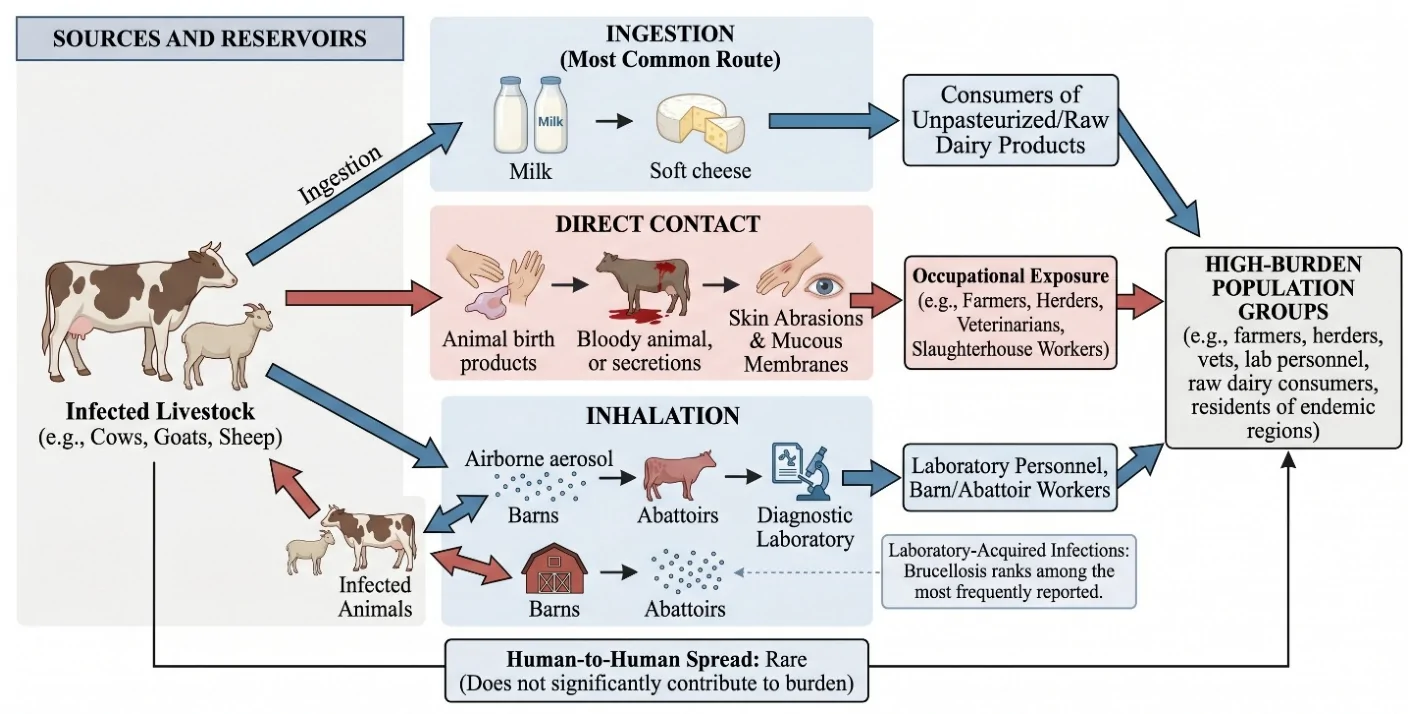
Transmission routes of brucellosis from infected livestock to high-burden human populations. Infected livestock transmit *Brucella* to humans through three main routes. Ingestion (the most common route) occurs via consumption of unpasteurized/raw dairy products such as milk and soft cheese. Direct contact with animal birth products, blood, secretions, or via skin abrasions and mucous membranes poses an occupational risk to farmers, herders, veterinarians, and slaughterhouse workers. Inhalation of airborne aerosols in barns, abattoirs, and diagnostic laboratories affects laboratory and abattoir personnel, with laboratory-acquired infections being among the most frequently reported. These routes converge on high-burden groups, including farmers, herders, veterinarians, laboratory staff, raw-dairy consumers, and residents of endemic regions. Human-to-human transmission is rare and does not significantly contribute to disease burden. The figure is an original schematic created by the authors using BioRender.com.

Transmission to humans occurs through several overlapping routes (Figure 1). The most common is consumption of unpasteurized milk and dairy products such as soft cheeses, while direct contact with infected animals, their birth products, blood, or secretions allows entry through skin abrasions and mucous membranes [9]. Inhalation of contaminated aerosols is an important route in barns, abattoirs, and diagnostic laboratories, where brucellosis ranks among the most frequently reported laboratory-acquired infections; human-to-human spread is rare [10,11]. Consequently, the highest burden falls on occupationally exposed groups—farmers, herders, veterinarians, slaughterhouse workers, and laboratory personnel—as well as on consumers of raw dairy products and residents of endemic regions [4].

The persistence of brucellosis in both animal and human populations is largely attributable to the failure or incomplete implementation of national and regional eradication programs. A fundamental barrier to eradication is the economic impact of test-and-slaughter policies, in which confirmed seropositive animals must be culled, resulting in substantial losses for smallholder and commercial producers alike. In regions where compensation schemes are inadequate or absent, these economic pressures discourage reporting and compliance, perpetuating reservoir populations. In this context, a genomic understanding of host–pathogen interactions offer a critical pathway toward more targeted and economically viable control strategies—including the identification of host genetic resistance markers, the development of improved vaccines, and the characterization of pathogen virulence determinants that drive persistence and immune evasion. The variable course of infection and the differing outcomes across hosts are increasingly attributed to genetic factors on both sides of the host–pathogen interface: polymorphisms that shape host susceptibility and resistance, and genomic determinants that govern *Brucella* virulence, persistence, and antimicrobial response. Advances in whole-genome sequencing, functional genomics, and transcriptomics now enable systematic dissection of these determinants. This review therefore aims to synthesize current evidence on the genetic basis of brucellosis by (i) examining host genetic markers of susceptibility and resistance in humans and livestock, (ii) appraising transcriptomic signatures proposed as diagnostic biomarkers, (iii) summarizing pathogen virulence architectures, regulatory networks, and their interaction with host molecular machinery, (iv) evaluating antimicrobial resistance and its unconventional mechanisms, treatment challenges, and the genomic epidemiology of circulating strains, and (v) identifying key gaps and translational priorities for genetics-informed diagnosis, vaccine design, and control. Throughout, we foreground the veterinary and One Health dimensions: we prioritize evidence from cattle, goats, sheep, and buffalo, and we treat human data primarily as mechanistic context, whose findings require species-specific validation before they can inform veterinary practice.

## 2. Literature Search Methodology

This article is a narrative review and is reported with attention to the transparency principles of the SANRA (Scale for the Assessment of Narrative Review Articles) guidance; it is not a systematic review and was not registered as such. The aim was to synthesize and critically appraise, rather than exhaustively enumerate, the genetic determinants of brucellosis on both the host and pathogen sides.

Literature was identified by searching PubMed, Scopus, Web of Science and Google Scholar for records published between January 2015 and June 2026, with seminal earlier work included where relevant. Search terms combined the concepts of brucellosis and *Brucella* with genetic, genomic and functional descriptors, using Boolean strings such as (“brucellosis” OR “*Brucella*”) AND (“genetic susceptibility” OR “polymorphism” OR “GWAS” OR “transcriptomic” OR “virulence gene” OR “antimicrobial resistance” OR “non-coding RNA” OR “functional genomics”). Reference lists of retrieved articles were screened to identify additional sources.

Peer-reviewed original studies and reviews reporting host genetic markers, transcriptomic signatures, pathogen virulence and regulatory determinants, or antimicrobial-resistance mechanisms in brucellosis met the inclusion criteria. Non-English records without an English abstract, conference abstracts without full data, and studies unrelated to genetic determinants were excluded. Because this is a narrative synthesis, no formal risk-of-bias scoring was applied; instead, the strength of evidence for each marker is appraised qualitatively throughout the text and summarized using a structured evidence-grading scheme (Table 1), graded from association-only findings through to field or clinical validation.

## 3. The Genetic Complexity of Brucellosis: Why Genetics Matters Now

Despite decades of research, fundamental questions persist: why do some individuals clear infection while others progress to chronicity? Why do genetically similar *Brucella* strains exhibit variable virulence in different hosts? And why is AMR emerging in a genus that historically carried no classical resistance determinants? The answers to these questions are increasingly being sought at the genetic level, driven by the convergence of affordable sequencing technologies, computational biology, and functional genomic tools.

The past five years have witnessed substantial recent growth in the identification of brucellosis-associated genetic markers. On the host side, candidate gene studies and genome-wide association analyses have implicated polymorphisms in innate immune receptors, cytokine genes, and immunomodulatory molecules in differential susceptibility across humans, goats, cattle, sheep, and buffalo [12,13,14]; however, most candidate-gene studies are constrained by small sample sizes, single-population designs, and a lack of independent replication, which limits the reliability of reported effect sizes. On the pathogen side, transposon sequencing (Tn-seq, TraDIS), CRISPR screens, and comparative genomics have moved beyond cataloging virulence genes to interrogating their context-dependent essentiality [15,16,17]. Meanwhile, transcriptomic profiling combined with machine learning has generated candidate diagnostic biomarker panels with promising clinical performance [18,19,20]; however, many of these studies reuse the same public datasets for both model training and internal validation without independent external cohorts, raising concerns about overfitting and limited generalizability.

Yet this burgeoning literature is fragmented across host species, *Brucella* species, and methodological platforms, making it difficult to discern which findings represent robust, translatable markers versus statistical associations lacking biological plausibility. This review aims to impose analytical coherence on this landscape. Rather than cataloging individual studies, we organize the evidence thematically—host susceptibility determinants, transcriptomic diagnostic signatures, pathogen virulence architectures, resistance mechanisms, and non-coding RNA regulators—and critically evaluate the strength of evidence, mechanistic depth, and translational potential at each level. We conclude by identifying the most consequential gaps and proposing priorities for the next phase of research.

## 4. Host Genetic Determinants of Susceptibility and Resistance

### 4.1. Human Genetic Associations

Human genetic studies of brucellosis susceptibility have relied primarily on candidate-gene approaches targeting the innate immune pathway. Mahmoudi et al. [12] reported associations between vitamin D receptor (VDR) polymorphisms and brucellosis risk, with the ApaI aa genotype conferring 53-fold and the FokI ff genotype 37-fold increased odds of disease [12]; these estimates derive from a single case–control study and should be interpreted with caution pending reporting of sample size, genotype frequencies and confidence intervals. The TNF -238 promoter polymorphism (rs361525) represents a more conservative and potentially prognostic association, pending independent validation, since the minor A allele independently predicted brucellar spondylodiscitis after adjustment for host variables [21]. No comparable effect was observed for *TLR4* polymorphisms, underscoring both the selectivity of genetic effects and the limitation of testing only a few loci. A critical limitation across these studies is the absence of functional validation: statistical associations, however significant, do not establish causation. The field needs reporter assays, gene-edited cell lines, and ex vivo studies showing that risk alleles alter protein expression, ligand binding, or downstream signaling during *Brucella* infection. Without such evidence, their translational value remains speculative.

Multiple meta-analyses have further clarified the landscape of cytokine gene polymorphisms in human brucellosis. Zafari et al. [22] identified *IL-4* (rs2243250) and *IL-18* (rs1946519) mutant alleles as susceptibility markers, while *IFN-gamma* UTR5644, *TGF-beta* rs1800470/rs1800471, *TNF-alpha* rs1800629, and *IL-10* rs1800872 showed protective associations [22]. Similarly, Fu et al. [23] linked the *TGF-beta1* codon 25 GG genotype to increased risk but found no association for *IL-6*, *IL-10*, or *TGF-beta1* codon 10 polymorphisms [23]. In Iranian cohorts, Sepanjnia et al. [24] found the *TGF-beta1* +868 TT genotype and TT/GG diplotype conferred susceptibility [24], and Eskandari-Nasab et al. [25] identified the IFN-gamma +874 AA genotype as a risk factor [25]. Lou et al. [26] provided rare functional validation: *TNFAIP3* TT > A variants (rs148314165/rs200820567) protected against brucellosis (OR = 0.34) via reduced *TNFAIP3* expression, enhanced NF-κB signaling, and elevated *IL-10*, *IL-6*, and *IL-1beta* production in *Brucella abortus*-stimulated monocytes—showing that variants linked to autoimmune disease risk can paradoxically protect against intracellular pathogens [26].

*TNF* and *IL1B* consistently emerge as central early-response genes in macrophages during *Brucella* infection, upregulated and enriched in immune pathways under Bcl3 regulation [27]. It should be noted that these transcriptional responses reflect infection-induced expression rather than inherited susceptibility, and the two should not be conflated. IFN signaling is a key molecular signature of active brucellosis [28], with IFN-related genes downregulated after successful treatment [29].

### 4.2. Livestock Genetic Markers: Toward Marker-Assisted Selection

Two caveats frame this livestock evidence. First, associations reported in humans cannot be extrapolated directly to animals: *Brucella* host responses are species-specific, and reservoir species, infecting doses, and immune architecture differ across hosts. Second, none of the markers below is yet ready for routine marker-assisted selection, and each requires independent replication and field validation in the target breed before it could be used in breeding programs. In livestock species, the translational goal differs fundamentally: rather than individual risk prediction, the objective is to identify genetic variants that can be incorporated into breeding programs for disease resistance only after replication and field validation. Here, the evidence base is broader but methodologically heterogeneous. In Shaanbei white cashmere goats, *ASAP1* insertion/deletion polymorphisms at the P2 and P7 loci are significantly associated with *Brucella* resistance, with resistant haplotypes (Hap3, Hap5) showing higher ASAP1 expression and more rapid pro-inflammatory cytokine activation upon LPS stimulation [30]. This study is notable for moving beyond genotype–phenotype association to demonstrate a plausible functional mechanism. In contrast, *CTLA4* InDel polymorphisms were associated with increased susceptibility to brucellosis under codominant and dominant genetic models, consistent with *CTLA4*’s established role as a negative regulator of T-cell activation [31]. This association marks a correlation rather than a demonstrated causal effect, yet it aligns with the biological expectation that impaired down-regulation of T-cell responses shapes host control of intracellular *Brucella.* Importantly, the null result for *TNF* and *PTPRT* InDels in the same goat breed reported by Zhang et al. underscores that not all immune gene polymorphisms influence brucellosis outcomes. Such negative findings are as informative as positive associations for refining candidate gene selection, since they help exclude loci that carry no measurable effect and focus attention on markers with genuine discriminatory value [32].

The sole genome-wide association study in goats identified a significantly associated locus on chromosomes 15 and 23, including candidate immune-related genes, such as *ZAP70*, *IL2RB*, and *NAIP* [14]. While the GWAS design offers unbiased genomic coverage, the modest sample size limits statistical power, and the study does not report genomic inflation factors, correction for population structure, or genome-wide significance thresholds, nor has the association been independently replicated. In Italian Mediterranean buffalo, a *TLR2* coding variant (c.374T) in exon 2 was associated with brucellosis resistance (OR = 0.61), supported by in silico predictions of altered protein activity [13]. This finding aligns with TLR2’s known role in recognizing *Brucella* lipoproteins (Omp10, Omp16, Omp19), providing mechanistic coherence. In sheep, a whole-genome sequencing approach in an F2 population identified 205 candidate SNPs enriched in adherens junction and Hippo signaling pathways [33], revealing that brucellosis resistance may involve mechanisms of cell adhesion and tissue integrity beyond classical immunity. Collectively, epigenetic modifications add another dimension: TLR5 promoter methylation levels were significantly higher in healthy than in *Brucella*-infected Saanen goats, independent of underlying SNP genotypes; however, whether these methylation differences were adjusted for cell-type composition, infection stage, age, environmental exposure, or production status was not reported [34].

In cattle, Prakash et al. evaluated 20 SNPs across cytokine and innate immunity genes, identifying significant associations at SLC11A1 (+1066 C/G), TLR1 (+1446 C/A, +1380 G/A), and TLR4 (+399 C/T), with the TLR4 CT genotype conferring notably high susceptibility [35]. Gene expression studies in infected cattle have provided additional mechanistic context: Priyanka et al. [36] documented a shift from Th1- to Th2-dominant cytokine profiles during chronic bovine brucellosis, with enhanced IFN-gamma, IL-1beta, and IL-6 alongside reduced TNF-alpha and IL-4 [36], while Im et al. [33] showed that recombinant *B. abortus* proteins selectively induced IL-6, IL-12p40, and IFN-gamma in bovine PBMCs without triggering apoptosis [37].

In goats, additional candidate gene studies have expanded the repertoire of resistance-associated loci. Hasenauer et al. [38] linked TNF rs668920841 and an IFNGR1-proximal microsatellite (BMS2753) to susceptibility, while a heterozygous genotype at microsatellite INRA111 near VRK2 was associated with resistance [38]. Rossi et al. identified IRF3-540 (rs660531540) as a functional resistance determinant, with the deletion extending an upstream open reading frame and potentially reducing translation efficiency [39], and Rossi et al. provided the first evidence linking PTPRT intron 8 polymorphisms to brucellosis resistance, with the TTCCT haplotype conferring strong protection [40]. Functional studies in goat models have also revealed novel mechanisms: Wang C et al. [18] demonstrated that BLOS1 overexpression promotes NF-κB /TLR4 activation, autophagy-mediated bacterial clearance, and intestinal microbiota remodeling following *Brucella* LPS challenge [18], while Gemini et al. characterized stage-specific immune signatures distinguishing persistent from latent infection, with persistent infection showing elevated pro-inflammatory responses and latent infection characterized by IL-10 upregulation and antioxidant defense activation [41].

This observation suggests that brucellosis susceptibility is determined not only by DNA sequence variation but also, potentially, by epigenetic states that may be environmentally modifiable—a finding with implications for both diagnostics and husbandry interventions (Table 2).

## 5. Transcriptomic Signatures as Diagnostic Biomarkers

The application of transcriptomic profiling to brucellosis diagnostics represents one of the most rapidly advancing areas in the field. Multiple independent studies have leveraged public gene expression datasets—predominantly GSE69597—along with machine-learning feature-selection algorithms to identify hub gene signatures that distinguish patients with brucellosis from healthy controls and, in some cases, acute from chronic disease. Consistently, Wang et al. [42] identified six hub genes (*RTP5*, *KIF19*, *CDKN2A*, *RCAN2*, *GLB1L3*, and *IL12RB2*) using LASSO, Gaussian mixture models, and SVM-RFE, with individual AUC values ranging from 0.735 to 0.781 (indicating moderate to good, rather than clinically definitive, discrimination) [42]. Furthermore, Yuan et al. [19], analyzing the same dataset but filtered through 1793 immune-related genes, identified four candidates (*RFX5*, *FYN*, *IL12RB2*, and *LGR6*), with *IL12RB2* and *FYN* validated by qRT-PCR in independent clinical cohorts. The convergence on *IL12RB2* across these independent analyses supports further investigation of *IL12RB2*: as a subunit of the IL-12 receptor driving Th1 differentiation and *IFN-γ* production, its dysregulation directly reflects the central immunological axis disrupted during brucellosis [19]. Similarly, Hu et al. [20] identified *CDK1*, *MAPK11*, and *PDIA3* through gradient boosting and random forest models, with validation in 88 patients by flow cytometry, RT-qPCR, and Western blot [20]. Wang et al. [43] took a different angle by focusing on prosaposin-related genes, identifying *SKAP2*, *EIF2B1*, *PRKAB1*, *IRF8*, and *RPN1*, achieving an AUC of 0.844 and validating by qPCR in an independent cohort [43].

Several patterns emerge from these studies. First, the heavy reliance on a single public dataset (GSE69597) raises concerns about overfitting and dataset-specific biases; independent multi-center validation cohorts are essential before any signature can be considered clinically robust. Second, while machine learning algorithms excel at feature selection, they are agnostic to biological mechanisms—a gene may be diagnostically useful without being pathogenically relevant. Third, the diversity of identified gene panels across studies that use the same dataset but different analytical pipelines illustrate how sensitive feature selection is to algorithmic choices and pre-filtering strategies. This is not necessarily a weakness—different pipelines may capture complementary aspects of the transcriptomic response—but it complicates the path toward a consensus diagnostic panel. It should be stressed that these signatures are discovery-stage research markers rather than established diagnostic tests, and that panels derived from human cohorts cannot be assumed to translate to cattle, goats, sheep, or buffalo, where the transcriptomic response to *Brucella* may differ substantially and require dedicated species-specific validation.

Disease staging represents a particularly valuable application. Çağan et al. proposed that directional changes in *ABI3*, *CLEC12B*, *PPP2R4*, *DDIT4L*, *PIAS4*, and *IDO* were candidate markers for distinguishing acute brucellosis from treatment response and potentially indicating chronic progression [44]. The single-cell RNA sequencing atlas of Wang et al. [45]—encompassing 290,369 cells from 35 individuals, including 29 brucellosis patients across acute (*n* = 10), sub-acute (*n* = 9), and chronic (*n* = 10) phases plus six healthy donors—provides the most granular view yet, identifying S100A8/A9-associated inflammatory signatures in acute disease and LAG3/CTLA4-mediated T-cell exhaustion in chronicity. This resolution reveals cell-type-specific mechanisms that are invisible to bulk transcriptomics and positions single-cell approaches as a promising avenue for biomarker discovery. It should be emphasized that none of the transcriptomic signatures discussed above constitutes a validated diagnostic test; all require prospective, multi-center validation before clinical use [45].

In livestock, gene expression studies have focused on understanding pathogenesis rather than on diagnostics themselves. Mabrouk et al. [46] demonstrated concurrent *NOD2* upregulation and *TLR9* downregulation in chronically infected cattle, suggesting that *Brucella* actively suppresses endosomal DNA recognition while triggering compensatory cytoplasmic sensing [46]. Elsayed et al. [47] documented comprehensive immune gene dysregulation in naturally infected Shami goats, with upregulation of innate immune receptors (*TLR1*, *TLR9*, *CARD15*, *IRF3*) alongside downregulation of antioxidant defenses (*GPX1*, *NQO1*, *Nrf2*), suggesting that oxidative stress vulnerability may compound immune dysfunction during infection [47].

Earlier transcriptomic analyses provided foundational insights into host–pathogen gene expression dynamics. Hop et al. employed dual RNA-seq to profile intracellular *B. abortus* and murine macrophages simultaneously, revealing that over 25% of bacterial genes were upregulated during intracellular growth, primarily those involved in secretion and protein biosynthesis, while host macrophages mounted robust pro-inflammatory responses through *TNF*, *IL-1β*, *IL-6*, and NF-κB signaling [48]. At the post-transcriptional level, Rong et al. demonstrated that CD14 knockdown in *B. melitensis*-infected macrophages altered miRNA expression profiles, with upregulation of miR-199a-3p and miR-183-5p leading to downregulation of the E3 ubiquitin ligase Cbl-b, implicating miRNA-mediated regulation as an additional layer of control in host–pathogen interactions [49].

## 6. Pathogen-Side Genetic Determinants: From Catalogs to Context-Dependent Essentiality

### 6.1. The Core Virulence Architecture

Comparative genomic studies across hundreds of *Brucella* isolates consistently identify two pillar systems: the type IV secretion system (T4SS, virB operon) and lipopolysaccharide (LPS) biosynthesis machinery. These are near-universally conserved, with 42 VFDB virulence genes present in >99% of 275 *B. melitensis* genomes [50]. Studies from India [51], Tunisia [52,53], Kazakhstan [1], Greece [54], and China [55] consistently report the conservation of these core virulence determinants, reinforcing their essentiality and supporting their prioritization for further investigation of vaccines and antimicrobial targets. Notably, the preliminary in silico analysis by Özgen et al. [56] identified VirB5 (extracellular) and VirB7 (outer membrane) as subunit vaccine candidates based on subcellular localization, while lpxC’s strong connectivity in protein interaction networks highlights it as a potential antimicrobial target [56].

However, this apparent conservation masks biologically significant variation. The *virB10* gene—encoding a critical T4SS component—shows high variability among *B. abortus* strains [57] and was consistently absent from three *B. abortus* strains on the Qinghai–Tibet Plateau, along with *bmaA* and *btpB* [58]. This triple-gene absence in a geographically defined lineage raises the hypothesis that these strains may represent attenuated variants or may have evolved alternative virulence mechanisms adapted to high-altitude hosts, although this remains unproven. SNP analysis further reveals species-specific mutation patterns: BetB, PrpA, and MviN polymorphisms occur exclusively in *B. melitensis*, while Ure mutations are restricted to *B. abortus* [1,50], suggesting species-specific evolutionary pressures on virulence gene function.

Although *B. melitensis* is classically associated with small ruminants (sheep and goats), there is accumulating evidence of its detection in cattle and water buffaloes in endemic regions, most notably in the Middle East, the Mediterranean basin, and South and Southeast Asia [11,59,60,61,62]. In cattle, *B. melitensis* has been isolated in mixed-farming systems where small ruminants and bovines share grazing areas or housing, suggesting inter-species transmission facilitated by close contact [11,60,63,64]. In water buffaloes (*Bubalus bubalis*), serological and culture-confirmed infections with *B. melitensis* have been reported in countries where buffalo husbandry and small ruminant farming frequently overlap [65,66]. In wildlife, spillover of *B. melitensis* from domestic small ruminants to Iberian ibex (*Capra pyrenaica*) and other wild ungulates has been well documented in parts of Europe and Africa, raising concerns about a wildlife reservoir that could impede eradication [67,68,69,70]. These observations support the plausibility of a sustained “close cycle” of *B. melitensis* transmission across multiple sympatric host species. The genomic characterization of *B. melitensis* strains isolated from non-classical hosts, and their comparison with small ruminant-derived strains using whole-genome sequencing and phylogenomic analyses, would help clarify the directionality of transmission and assess the risk these strains pose to human infection. Host-specific genomic adaptations in strains recovered from cattle or wildlife—if identified—would have significant implications for understanding host tropism and for the design of cross-reactive vaccine strategies.

Regional virulence gene prevalence studies have substantiated the conservation of core determinants while revealing host-association-specific variation. Hashemifar et al. found that seven key virulence genes (*btpA*, *btpB*, *virB5*, *vceC*, *bpe275*, *bspB*, *virB2*) were present in 100% of Iranian *Brucella* isolates, while *prpA* and *betB* showed differential distribution between human and animal sources [71]. Similarly, Naseri et al. reported 100% prevalence of ten virulence-associated genes across 57 *B. melitensis* clinical isolates from Iran, including the intracellular pathogenesis-related genes virB, znuA, and wbkA [72].

Beyond cataloging, functional characterization has revealed novel virulence determinants. Tian et al. [56] identified vhpA (bab_RS22045) as a small, highly conserved gene essential for *B. abortus* virulence in mice by regulating downstream gene networks, with functional specificity demonstrated by the failure of homologs from Sinorhizobium meliloti and Agrobacterium tumefaciens to restore virulence [73]. Jung et al. [57] showed that mutation of the ABC transporter permease BruAb2_1031 enhanced intracellular survival by suppressing host cytokine and apoptosis responses [74], and Zhou D et al. [58] demonstrated that deletion of outer membrane protein Omp16 in B. suis upregulated 52 immune-related genes including *IL-6*, *IL-12beta*, *CCL2*, and *CCL22* in macrophages, indicating that Omp16 functions to actively suppress proinflammatory signaling during infection [75]. Consistent with this theme of active immune modulation, the T4SS effectors *BPE005*, *BPE275*, and *BPE123* induce Th1/Th2 cytokine responses without triggering host–cell apoptosis [76], while the *LuxR*-family transcriptional regulator *VjbR* directly controls genes required for early intracellular survival [77].

On the translational front, proteomic analyses identified PGLYRP2, F2, and GSN as high-performance biomarkers distinguishing acute from chronic brucellosis [78]. Genomic surveillance in Egypt identified vaccine-associated *B. abortus* strains and putative AMR determinants (e.g., *ErmC*, *TetK*, reported as database annotations whose functional status, chromosomal versus mobile-element origin, and possible contaminant nature remain unconfirmed) in *B. melitensis* [79], whereas field isolates from South Africa exhibited high multiple antibiotic resistance (MAR) indices, with doxycycline and tetracycline resistance observed in goat and sheep isolates [80].

### 6.2. Functional Genomics: Context-Dependent Essentiality

The most conceptually significant advance in pathogen-side genetics is the demonstration that *Brucella* gene essentiality is not fixed but context-dependent. Potemberg et al. [67] showed that LPS biosynthesis genes essential for lung survival in wild-type mice become dispensable in IL-17RA-deficient mice, while fatty acid β-oxidation genes (fadA, fadJ) are no longer required in asthmatic mice. This finding fundamentally reframes our understanding: the virulence gene repertoire is not a static toolkit but a dynamic system whose functional requirements are dictated by the host immune environment [81]. Barbieux et al. [14] extended this concept through Tn-seq in mouse spleens and lungs, identifying 87 genes essential in both organs, 170 spleen-specific genes, and 48 lung-specific genes, with *trpD* essential only in lungs and *znuA* only in spleens [15].

TraDIS screening by Jiang et al. identified 374 genes essential for macrophage survival, with functional validation confirming roles for membrane genes (cydDC), metabolic genes (*ptsP*), and regulatory genes (*BME_RS00125*) [16]. The CRISPR screen by Xu et al. approached the same question from the host side, identifying DEFB103B and other host genes whose knockout alters intracellular *Brucella* fate—thereby establishing that intracellular survival depends on joint host and pathogen genetic factors [17].

### 6.3. Regulatory Networks Governing Virulence

A rapidly expanding body of work is mapping the transcriptional regulatory networks that coordinate the expression of Brucella virulence genes. These regulators can be organized into three functional tiers: global transcriptional regulators, stress-response regulators, and regulators of virulence-associated metabolism and trafficking. At the broadest level, transcriptional architecture is dominated by *MucR*, an H-NS-like global silencer that occupies 546 ChIP-seq binding peaks and represses adhesins and quorum-sensing regulators until dedicated counter-silencers relieve this repression [82].

A second tier is dedicated to sensing host-imposed stress. GntR8 directly activates *clpP* to mount oxidative-stress defense, and its deletion attenuates both macrophage survival and virulence in mice [83]. Metal availability, in turn, is monitored by Fur4 and Mur—redundant, metal-dependent repressors of the virB operon—which compete with the transcriptional activators *IHF*, *HutC*, *MdrA*, and *VjbR* to couple intracellular metal limitation to finely tuned T4SS expression during intracellular infection [84].

A third-tier tunes virulence-associated metabolic and trafficking programs. BvtR governs nitrogen metabolism and siderophore biosynthesis and is required for full virulence in mice [85], whereas DeoR1 modulates the intracellular-trafficking genes essential for pathogenesis [86]. Collectively, these studies reveal a multi-layered regulatory architecture in which virulence gene expression is not constitutive but exquisitely tuned to environmental cues—a sophistication that may underlie Brucella’s remarkable capacity for chronic persistence (Table 3).

## 7. Antimicrobial Resistance in *Brucella*: Metabolic Architectures, Surveillance Gaps, and Clinical Failure

Perhaps the most intellectually challenging area in *Brucella* genetics is AMR, which defies the paradigms established in other bacterial pathogens. Classical AMR genes—acquired β-lactamases, aminoglycoside-modifying enzymes, mobile resistance elements—are often not detected in *Brucella* genomes, even when phenotypic resistance is observed [2,51,52,53]. The detection of database-annotated determinants such as ErmC and TetK in *B. melitensis* (Section 6) does not contradict this general pattern, as their functional significance, genomic context, and possible contaminant origin remain unverified. Instead, resistance appears to operate through metabolic and regulatory mechanisms that alter the intracellular environment.

Three recent discoveries exemplify this unconventional architecture. Geng et al. [65] demonstrated that *hflX*, encoding a ribosome-associated GTPase, mediates rifampicin resistance through coordinated regulation of RNA polymerase subunits (*rpoA*, *rpoB*, *rpoC*) and sigma factors (*rpoD*, *rpoH*), without altering cell envelope integrity [87]. Conversely, Yuan et al. [88] showed that STPK deletion in *B. melitensis* increases rifampicin resistance (assessed by broth microdilution) by upregulating glutathione synthesis and decreasing NADPH oxidase activity in an in vitro macrophage infection model, thereby enhancing antioxidant capacity against rifampicin-induced reactive oxygen species. These two findings—one gene whose loss increases susceptibility, another whose loss increases resistance, both affecting the same antibiotic—illustrate the complexity of resistance circuitry in *Brucella* [88]. Yang et al. [89] identified yet another mechanism: the glycoside hydrolase GH25, characterized in clinical *B. melitensis* isolates by combining gene-deletion mutagenesis with metabolomic profiling, mediates trimethoprim-sulfamethoxazole resistance through altered energy metabolism and protein interactions with XylF, with high-MIC and low-MIC isolates activating distinct metabolic pathways under antibiotic pressure [89].

The universal presence of efflux-related genes (*mprF*, *bepC–G*) across all sequenced isolates, regardless of resistance phenotype [53,90,91], suggests that efflux pump genes are baseline genomic features that may nonetheless require expression, regulation or mutation data to interpret their contribution to phenotypic resistance, rather than acquired determinants alone. The observation that mutations in *rpoB*, *gyrA*, and *folP* occur in both resistant and susceptible isolates [91,92] further undermines simple genotype–phenotype correlations and argues for a multifactorial, systems-level understanding of *Brucella* AMR that integrates transcriptomic and proteomic data. Interpretation of the studies discussed here is further constrained by heterogeneous susceptibility-testing methods; where reported, phenotypic resistance was assessed by broth microdilution or Etest and interpreted against CLSI/EUCAST breakpoints (or, in their absence, epidemiological cut-off values), and all live-culture work requires biosafety level 3 containment. Reported MIC ranges and the specific interpretive criteria applied are inconsistently documented across the studies reviewed, limiting direct comparability of resistance phenotypes. Standardized MIC methods, defined breakpoints and consistent reporting are needed before genotype–phenotype correlations can be established. Accordingly, the resistance mechanisms described here should be regarded as biologically informative rather than diagnostically actionable: none is yet validated for predicting clinical resistance, and *Brucella* genotype cannot at present be used to guide antimicrobial treatment.

Although *Brucella* species are not classically considered high-priority AMR pathogens, the emergence of reduced susceptibility to first-line agents (doxycycline, rifampicin, and trimethoprim-sulfamethoxazole) in clinical and veterinary isolates warrants attention [93,94]. Antibiotic pressure arising from both human therapeutic use and, critically, from the widespread prophylactic and therapeutic use of tetracyclines and sulfonamides in livestock production systems, represents a plausible driver of resistance evolution in *Brucella* populations circulating at the livestock–human interface [65,95]. Whole-genome sequencing studies have identified mutations in rpoB (associated with rifampicin resistance) and upregulation of efflux pumps as potential mechanisms of reduced susceptibility in human isolates [91,96].

In low-prevalence countries—including many high-income northern European nations, North America, and Australia—brucellosis is predominantly encountered as an imported or travel-associated infection, and isolates from these settings are infrequently subjected to systematic AMR surveillance or genomic characterization [9,53,97,98]. This represents a significant gap in our understanding, as travelers and migrants from highly endemic regions may carry strains with distinct resistance profiles that are not captured by surveillance systems focused on endemic countries [99,100].

Of particular clinical concern are cases of therapeutic failure in human brucellosis, which may result from non-compliance with prolonged treatment regimens, reinfection, relapse due to intracellular persistence of *Brucella*, or genuine AMR. Genomic studies of human clinical isolates from cases of relapse or treatment failure have the potential to distinguish between these mechanisms and identify actionable determinants of resistance. At present, such studies remain limited in number and geographic scope, and prospective collection and sequencing of clinical isolates from treatment-failure cases should be considered a research priority.

## 8. Non-Coding RNA Regulators and Host–Pathogen Crosstalk

An emerging frontier in brucellosis genetics involves non-coding RNAs—both host long non-coding RNAs (lncRNAs) and bacterial antisense RNAs—as regulators of host–pathogen interaction. Liu G et al. demonstrated that *Brucella* outer membrane protein Omp19 reprograms the host lncRNA landscape, with 645 differentially expressed lncRNAs identified. Among these, *MIR99AHG* functions as a positive regulator of inflammation, upregulating *IL-1β*, *IL-6*, *IL-8*, *IFN-α*, *IFN-β*, and *TNF-α* while suppressing IL-10 [101]. Kodori et al. reviewed broader evidence implicating IFNG-AS1 and NLRP3-associated lncRNAs as upregulated during brucellosis, with Gm28309 downregulated in a manner that facilitates immunosuppression and bacterial survival [102].

On the pathogen side, Munoz-Bucio et al. identified asRNA_0067, a cis-encoded antisense RNA whose expression is induced by intraphagocytic stressors and whose deletion reduces virB2 transcription and bacterial survival. This finding reveals a previously unsuspected layer of post-transcriptional regulation governing T4SS expression within the host [103]. From the host innate immune sensing perspective, Gomes et al. (74) demonstrated that ZBP1 acts as a cytosolic DNA sensor contributing to IFN-β expression during *B. abortus* infection, working redundantly with the STING pathway [104].

The functional consequences of pathogen effectors on host cell signaling are being increasingly resolved. Khonsari et al. showed that OMP10 modulates BCAP31, CASP8, and IL1B to orchestrate ER stress, apoptotic priming, and inflammatory signaling [105]. Wu et al. identified the TYROBP–SQSTM1–NF-κB axis as a key signaling pathway in macrophage responses to *Brucella* LPS [106]. Zhao et al. identified glutaminase (Gls) as a critical NF-κB target gene that regulates M1/M2 macrophage polarization switching during infection [107]. Meanwhile, the identification of novel T4SS effectors RS15060 and RS10635 [108], alongside the functional characterization of BPE005, BPE275, and BPE123 as Th1/Th2 cytokine inducers [86], continues to expand the catalog of molecular mediators at the host–pathogen interface. Hu et al. further demonstrated that *Brucella* spondylitis is driven by CXCL8/CCL3/CCL4L2 via TLR4 signaling, thereby distinguishing it molecularly from tuberculous spondylitis, which is mediated by HLA-DRB genes and T-cell differentiation pathways [109].

## 9. Integrative Perspectives and Outstanding Questions

This review reveals a field at an inflection point. The descriptive phase—identifying which genes are differentially expressed, polymorphic, essential, or associated with disease—has been remarkably productive. However, several critical gaps impede the translation of genetic catalogs into clinical and veterinary applications.

A central limitation is the mechanistic gap, as the vast majority of host genetic associations lack functional validation. Statistical significance in a case–control study does not explain how a polymorphism alters protein function, immune cell behavior, or infection outcome. The field requires the systematic integration of genetic association with CRISPR-based functional studies in relevant cell types, as exemplified by the *ASAP1* haplotype-function correlation in goat PBMCs [30] and the CRISPR screen that identified DEFB103B’s role in autophagy-mediated bacterial clearance [17]. Compounding this is a validation gap: transcriptomic biomarker studies overwhelmingly derive from a single dataset (GSE69597), and even those with independent qPCR validation use small cohorts (16–88 patients). Multi-center, prospective validation studies with standardized protocols are therefore essential, and the diagnostic panels identified to date should be tested against existing serological and culture-based diagnostics in head-to-head comparisons across endemic settings with varying *Brucella* species, disease stages, and co-morbidities.

Equally pressing is the integration gap, as host and pathogen genetics are almost exclusively studied in isolation. The demonstration that host immune status determines which *Brucella* genes are essential [81] argues powerfully for integrated host–pathogen genetic analyses—dual RNA-seq, matched host genotyping and pathogen sequencing from the same clinical samples, and systems biology approaches that model the genetic dialogue between organisms. Closely related is the AMR prediction gap, where the disconnection between genotype and resistance phenotype in *Brucella* demands new predictive frameworks. Classical AMR gene databases are insufficient (though not without value) for this genus; instead, metabolic network models integrating transcriptomic, proteomic, and metabolomic data may be required to predict resistance phenotypes from genomic sequences. The discoveries of *hflX* [87], *STPK* [88], and *GH25* [89] as mediators of resistance suggest that *Brucella* AMR operates through metabolic reprogramming rather than through target modification or drug inactivation—a paradigm that requires entirely different surveillance tools.

Further constraining the field is the species-specificity gap, as most transcriptomic and host genetic studies have been conducted in humans or goats, with cattle, buffalo, and sheep comparatively underrepresented. The divergent transcriptomic responses observed between genetically selected and unselected Holstein cattle [110]—suggesting that dairy production selection may have compromised anti-*Brucella* immunity—highlight that genetic background profoundly shapes host response and that findings from one species or breed cannot be uncritically extrapolated to another. Finally, a gap in non-coding RNA research persists: while the identification of lncRNAs (MIR99AHG, IFNG-AS1) and antisense RNAs (asRNA_0067) as regulators of brucellosis pathogenesis is exciting, functional characterization remains superficial. Loss-of-function studies in relevant infection models—not merely overexpression in cell lines—are needed to establish whether these non-coding RNAs represent viable therapeutic targets or merely correlated markers.

Limitations of the evidence base. Beyond these thematic gaps, several constraints temper the strength of the evidence synthesized here. Many host-genetic associations rest on single case–control studies with modest sample sizes, incomplete reporting of effect sizes and confidence intervals, and limited correction for population structure, so independent replication is frequently lacking. The reported associations are overwhelmingly statistical, with only a minority (for example, *ASAP1*, *TNFAIP3*, and *IRF3*) supported by functional experiments. Transcriptomic biomarker studies depend heavily on a single public dataset (GSE69597) and small validation cohorts, raising the risk of overfitting. Veterinary evidence is unevenly distributed across species, with goats and cattle better characterized than sheep, buffalo, dogs, and wildlife, and a bias toward publishing positive associations is likely. These limitations should be weighed when interpreting any individual marker and explain why most findings remain at the discovery stage.

## 10. Conclusions

The genetic determinants of brucellosis span a remarkable range—from single nucleotide polymorphisms in host immune receptors to global regulatory circuits in the pathogen, from protein-coding virulence genes to non-coding RNA regulators. The convergence of high-throughput sequencing, functional genomics, and computational biology has transformed this from a data-poor to a data-rich field in under a decade. Yet the transition from descriptive genetics to mechanistic understanding and clinical application remains incomplete. We propose that the most impactful next steps are threefold. First, multi-center prospective studies validating transcriptomic diagnostic panels (particularly *IL12RB2*-centered signatures) against current diagnostic standards in diverse endemic settings. Second, systematic functional validation of host genetic associations using gene-edited primary cells and organoid models infected with clinically relevant *Brucella* strains. Third, integrated dual RNA-seq studies of matched host–pathogen pairs to map the genetic co-dependencies that determine infection outcome. The ultimate vision is a precision medicine framework for brucellosis: one in which host genotyping informs individual risk assessment, transcriptomic profiling guides diagnosis and staging, pathogen genomics predicts antimicrobial susceptibility, and both inform rational vaccine design. Discovery tools are increasingly available; the major challenge is now validation, standardization and implementation, together with the clinical and translational infrastructure to deploy them. For veterinary medicine specifically, this remains a field of marker discovery rather than field-ready application: at present no host genetic marker, transcriptomic signature, or resistance determinant is validated for routine use in animal breeding, surveillance, diagnosis, or treatment, and each will require independent, species-specific, and field-level validation before it can responsibly inform veterinary practice or control programs.

## Figures and Tables

**Table 1:** Evidence-grading scheme used in this review to appraise host and pathogen genetic markers, from association-only findings to translational readiness.

Evidence Level	Definition	Example Marker(s) in This Review
1. Association only	Single statistical association (case–control or candidate-gene) without replication or functional data	VDR, TNF -238, CTLA4
2. Replicated association	Association reproduced across independent cohorts or supported by meta-analysis	Cytokine polymorphisms (IL-4, IL-18, TGF-beta1)
3. Functional validation	In vitro or ex vivo evidence that the variant/gene alters expression, signalling or pathogen handling	TNFAIP3, ASAP1, IRF3-540
4. Animal-model validation	Effect demonstrated in an in vivo infection model (e.g., mouse, or livestock challenge)	virB operon, GntR8, MucR, plsC
5. Field/clinical validation	Marker tested in naturally infected field populations or clinical cohorts	Transcriptomic panels (qPCR-validated), TLR2 buffalo variant
6. Translational readiness	Validated, standardized and ready for diagnostic or breeding-programs implementation	None yet; all markers remain investigational

**Table 2:** Host genetic markers associated with susceptibility and resistance to brucellosis across species.

Gene/Locus	Host Species	Population/Phenotype	Association	Effect Estimate & Validation Status	Reference
*VDR* (FokI, TaqI, ApaI, BsmI)	Human	Case–control; brucellosis vs. controls	Associated with increased susceptibility to *Brucella* infection (immune regulation)	Large ORs (up to ~53-fold) from a single study; CIs/sample size not fully reported; association only	[12]
*TNF* (-238 G>A, rs361525)	Human	Patients with/without spondylodiscitis	Associated with spondylodiscitis and susceptibility to brucellosis	Adjusted association; single cohort; association only	[21]
*TLR2* (c.374T)	Buffalo	Italian Mediterranean river buffalo	Associated with resistance to brucellosis	OR = 0.61; supported by in silico prediction; replicated/functional support partial	[13]
*TLR5* (g.435C>T, g.690G>T, g.978A>G, g.1832A>G)	Goat	Saanen goats; infected vs. healthy	May influence flagellin recognition and infection risk (promoter methylation)	Epigenetic association; not adjusted for cell composition; association only	[34]
*ASAP1* (P2, P7 InDels)	Goat	Resistant vs. susceptible haplotypes	May influence innate immune responses; associated with resistance	Haplotype-function link (LPS-stimulated cytokines); functional validation	[30]
*CTLA4* (P1, P2 InDels)	Goat	Codominant/dominant models	Associated with increased brucellosis risk (T-cell regulation)	Genotypic association; association only	[31]
*ZAP70*, *IL2RB*, *NAIP*	Goat	GWAS, chromosomes 15/23	Regulate multiple immune pathways; associated with increased susceptibility	GWAS with modest sample size; limited power/replication; association only	[14]
*CTNNA3*, *NCAM1*, *YAP1*	Sheep	F2 population, WGS (205 SNPs)	Enriched in cell-adhesion and Hippo signalling pathways; context-dependent (resistance/susceptibility)	Pathway-level enrichment; direction context-dependent; association only	[33]

**Table 3:** Key *Brucella* pathogen genes associated with virulence, intracellular survival, AMR, and regulatory functions.

Gene(s)/System	Category	Function	Key Evidence	Evidence Level	Species	Reference
virB operon (virB1-virB12)	Virulence (T4SS)	Effector secretion, intracellular survival	Essential in macrophages and mice; *virB10* variable in *B. abortus*	Animal-model validation	*B. melitensis, B. abortus*	[1,50,57,58]
wbk/wbo/lpx clusters	Virulence (LPS)	LPS biosynthesis, immune evasion	Dispensable in IL-17RA-/- mice; essential in lungs	Animal-model validation	*B. melitensis*	[15,81]
*cydDC*, *BME_RS07715*	Virulence (Membrane)	Stress resistance, membrane integrity	Deletion attenuates intracellular survival	Functional validation (macrophage)	*B. melitensis*	[16]
*ptsP*	Metabolism	Phosphotransferase system, carbon utilization	Essential for intracellular proliferation	Functional validation (macrophage)	*B. melitensis*	[16]
*hflX*	AMR	Ribosome-associated GTPase	Deletion increases rifampicin susceptibility; downregulates *rpoABC*	Functional validation	*B. abortus*	[87]
*STPK*	AMR	Serine/threonine kinase, redox regulation	Deletion increases rifampicin resistance via *GSH* upregulation	Functional validation	*B. melitensis*	[88]
*GH25*	AMR	Glycoside hydrolase, cell wall metabolism	Deletion confers SXT resistance; alters energy metabolism	Functional validation (clinical isolates)	*B. melitensis*	[89]
*GntR8 -> clpP*	Regulatory	Transcriptional regulation of oxidative stress	Deletion impairs macrophage survival and mouse virulence	Animal-model validation	*B. abortus*	[83]
*Fur4/Mur*	Regulatory	Metal-dependent *virB* repression	Dual deletion derepresses T4SS in macrophages	Functional validation	*B. abortus*	[84]
*MucR*	Regulatory	Global gene silencer (H-NS-like)	Silences adhesins, phosphodiesterase; 546 ChIP-seq peaks	Functional validation	*B. abortus*	[82]
*DeoR1*	Regulatory	Transcriptional regulation of trafficking genes	Deletion attenuates virulence in macrophages and mice	Animal-model validation	*B. abortus*	[86]
*plsC*	Essential (metabolism)	Phospholipid biosynthesis	Deletion reduces persistence; proposed attenuated/vaccine candidate (in vivo protection not yet demonstrated)	Animal-model (attenuation)	*B. melitensis*	[15]

## Data Availability

No new data were created or analyzed in this study. Data sharing is not applicable to this article.
